# MangoBase: A Genomics Portal and Gene Expression Atlas for *Mangifera indica*

**DOI:** 10.3390/plants12061273

**Published:** 2023-03-10

**Authors:** Aynhoa Gómez-Ollé, Amanda Bullones, Jose I. Hormaza, Lukas A. Mueller, Noe Fernandez-Pozo

**Affiliations:** 1 Institute for Mediterranean and Subtropical Horticulture “La Mayora” (IHSM La Mayora-CSIC-UMA), 29010 Málaga, Spain; aynhoagomezart@gmail.com (A.G.-O.); amandabullones@uma.es (A.B.); ihormaza@eelm.csic.es (J.I.H.); 2 Department of Biochemistry and Molecular Biology, Universidad de Málaga (UMA), 29010 Málaga, Spain; 3 Boyce Thompson Institute, Ithaca, NY 14853, USA; lam87@cornell.edu

**Keywords:** mango, *Mangifera indica*, bioinformatics, genomics, gene expression, RNA-seq, database, bioinformatics tools, fruit ripening

## Abstract

Mango (*Mangifera indica* L.) (2n = 40) is a member of the Anacardiaceae family, which was domesticated at least 4000 years ago in Asia. Mangoes are delicious fruits with great nutritional value. They are one of the major fruit crops worldwide, cultivated in more than 100 countries, with a production of more than 40 million tons. Recently the genome sequences of several mango varieties have been released, but there are no bioinformatics platforms dedicated to mango genomics and breeding to host mango omics data. Here, we present MangoBase, a web portal dedicated to mango genomics, which provides multiple interactive bioinformatics tools, sequences, and annotations to analyze, visualize, and download omics data related to mango. Additionally, MangoBase includes a gene expression atlas with 12 datasets and 80 experiments representing some of the most significant mango RNA-seq experiments published to this date. These experiments study mango fruit ripening in several cultivars with different pulp firmness and sweetness or peel coloration, and other experiments also study hot water postharvest treatment, infection with *C. gloeosporioides*, and the main mango tree organ tissues.

## 1. Introduction

Mango (*Mangifera indica* L.) (2n = 40) is a member of the Anacardiaceae family, which was domesticated at least 4000 years ago in different regions of Asia [1]. Two ecogeographic races of mango have been distinguished: the Indian type, in subtropical regions, which produces monoembryonic seeds, and the Southeast Asian type, in tropical regions, which produces polyembryonic seeds [2]. Mangoes belong to *Mangifera*, a genus with between 45 and 69 species, of which 26 produce edible fruits [3]. Mangoes are delicious fruits with great nutritional value. Mango is one of the major fruit crops worldwide, ranking fifth in terms of production among perennial fruit crops worldwide, and currently cultivated in more than 100 countries, with a production of more than 40 million tons in 2021 (FAOSTAT). India, China, Thailand, Indonesia, and Mexico are the main mango producing countries, although this crop is cultivated in tropical and subtropical regions of all continents but Antarctica, including regions far from the equator, such as the Mediterranean basin in the south of Europe, the north of Africa, and the Middle East. Despite its great economical interest and popularity, until recently, very limited genomic resources and bioinformatic tools were available for mango. However, since 2020, the genome sequences of several mango varieties such as ‘Alphonso’, ‘Tommy Atkins’, ‘Irwin’, and ‘Carabao’ have been sequenced and published [3,4,5,6], and other mango genome sequences such as those from the ‘Hong Xiang Ya’ [7] and ‘Amrapali’ varieties have become available in the BIG Genome Sequence Archive database at CNCB-NGDC (https://ngdc.cncb.ac.cn/ (accessed on 8 March 2023)) and the Sequence Read Archive (SRA) at NCBI (https://www.ncbi.nlm.nih.gov/sra (accessed on 8 March 2023)), respectively. Additionally, other species of *Mangifera*, such as *Mangifera altissima*, and *Mangifera odorata* have also been sequenced [4]. All these genome sequences facilitate mango research, providing reliable gene sets and references for transcriptomics, comparative genomics, and genotyping.

Many studies have investigated gene expression in mango, mostly with a focus on studying genes involved in fruit traits of agronomic interest. Some examples are studies to better understand fruit ripening [5,8,9,10,11], peel coloration [5], peel cuticle [12], pulp sweetness [11] and firmness [10], the response to low-temperature storage [13], hot water treatments to reduce postharvest diseases [14], and infection with *Colletotrichum gloeosporioides* [15]. These expression experiments are very useful to understand the mentioned processes and the genes involved in them, and they provide a great resource to identify alleles from different mango varieties. However, many of these datasets are based on different transcriptome de novo assemblies which have their own identifiers and are not comparable between experiments, and, in many cases, these expression datasets are only available as raw reads deposited at the SRA.

Here, we present MangoBase (https://mangobase.org/ (accessed on 8 March 2023)), a web portal dedicated to mango genomics, which provides multiple interactive bioinformatics tools, sequences, and annotations to analyze, visualize, and download omics data related to mango, including a gene expression atlas with 12 datasets and 80 experiments analyzed based on the Tommy Atkins genome reference and linked to their gene references and annotations.

## 2. Results

MangoBase is based on EasyGDB [16] and includes multiple mango genomics data and bioinformatics tools to explore them.

### 2.1. Available Genomic Data

The downloads section in MangoBase provides genomic data such as genome, gene, and protein sequences, and annotations from the mango cultivars ‘Tommy Atkins’, ‘Alphonso’, and ‘Hong Xiang Ya’. Additionally, the genome section provides statistics of the genome assemblies and links to their publications and data in generalist repositories such as NCBI and the CNCB. Moreover, a consensus genetic map based on seven mapping populations is available for visualization in the map section, and the genome annotations can be explored in the integrated genome browser.

### 2.2. Tools

#### 2.2.1. Genome Browser

MangoBase has a genome browser based on JBrowse [17] where it is possible to explore the genome sequence and annotations of the mango genomes of ‘Tommy Atkins’ v4 (TA4), ‘Alphonso’ v2.1, and ‘Hong Xiang Ya’ v1.

**Figure 1:**
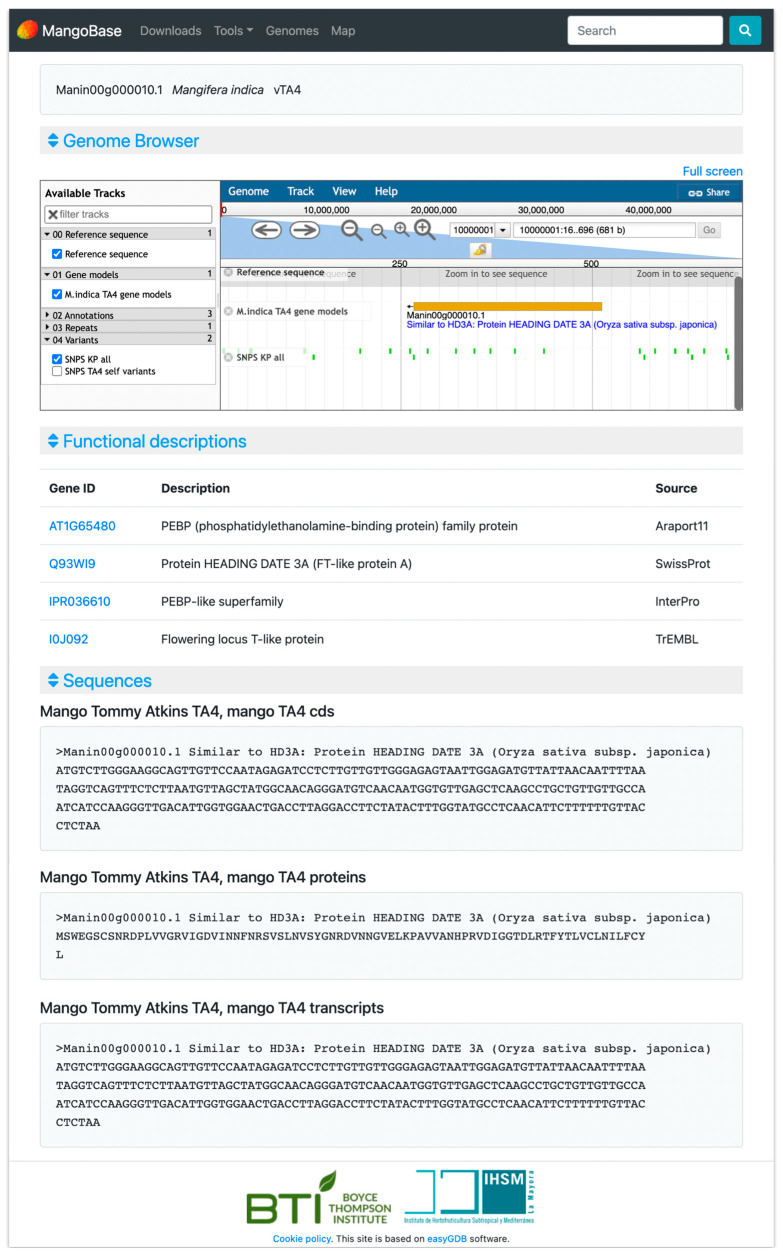
MangoBase gene page example.

The ‘Tommy Atkins’ genome browser contains tracks for the gene models, annotations with the gene structures predicted by Evidence modeler, and hits from BLASTn and BLASTx. In addition, another track displays the repeats identified by RepeatMasker, and others allow users to load genetic polymorphism data from a cross between ‘Tommy Atkins’ and ‘Kensington Pride’, and from a ‘Tommy Atkins’ self-pollinated population. The ‘Tommy Atkins’ gene models are connected with their annotations on the gene annotation page (Figure 1).

In the ‘Alphonso’ genome browser, tracks for the genome sequence and gene model annotations are available. The ‘Hong Xiang Ya’ genome is linked to the CNCB Genome Warehouse genome browser, where it contains similar tracks to ‘Alphonso’.

#### 2.2.2. Gene Annotation Search

MangoBase has dynamic gene pages with annotations and sequences of the genes from ‘Tommy Atkins’ genome v4 (Figure 1). These pages provide a frame of the genome browser showing the query gene, the gene sequences, and descriptions of the most similar sequences in Araport 11 (linked to TAIR) [18], SwissProt [19], trEMBL [19], InterPro [20] protein domains, and PlantCyc pathways and enzymes [21] (Figure 1). Araport 11 is the most recent annotation of Arabidopsis thaliana, the most important model plant species for functional genomics; Swissprot is a database of manually curated and reviewed proteins, and trEMBL comprehends a huge set of proteins that together with SwissProt cover all proteins in UniProt; InterPro combines several databases to classify protein domains by family; and PlantCyc provides plant metabolic pathways and genes with enzymatic activity that are involved in these pathways.

The search tool can find genes by their gene identifiers and by keywords from their functional descriptions, in the databases mentioned above. Search results are linked to the gene annotation pages and can be filtered, sorted, and downloaded in multiple formats.

#### 2.2.3. BLAST

The BLAST tool allows sequence similarity searching. The available datasets include the ‘Tommy Atkins’ and ‘Alphonso’ genomes, proteins, transcripts, and CDS (coding sequence of the transcripts).

The BLAST tool has the option of downloading the results in tabular format and provides a graphical visualization of the alignments of the best hits to the query gene. In the case of the ‘Tommy Atkins’ BLAST DBs, the BLAST results of proteins, transcripts, and CDS are linked to the gene pages, and to the genome browser in the case of the genome dataset. In the case of ‘Alphonso’ BLAST DBs, results are linked to the NCBI, except for the genome, which is linked to the genome browser in MangoBase.

#### 2.2.4. Sequence and Annotation Extraction Tools

The Sequence Extraction tool returns sequences in FASTA format for a provided list of gene names. ‘Tommy Atkins’ and ‘Alphonso’ protein, transcript, and genome sequences can be retrieved. Similarly, a list of gene identifiers can be provided to the Annotation Extraction Tool to obtain a table with the available annotations for those genes. In this case, the resulting table can be filtered, sorted by column, and downloaded in several formats such as CSV, Excel, PDF, or copied to the clipboard as a tab-delimited file. This feature facilitates the annotation of results from other experiments such as differential expression analyses. For example, just by pasting a gene ID list of differentially expressed genes in the Annotation Extraction tool, it is possible to obtain multiple annotations that can be easily pasted together with the differential expression analysis results. The resulting table has links to the MangoBase annotation page, and links and descriptions from the Araport 11 (linked to TAIR), SwissProt, trEMBL, InterPro protein domains, and PlantCyc pathways.

#### 2.2.5. Gene Lookup and Gene Enrichment Set Tools

A gene lookup tool allows researchers to easily identify the most similar genes between the available mango gene annotations from the varieties ‘Tommy Atkins’, ‘Alphonso’, and ‘Hong Xiang Ya’. In this tool, it is possible to convert gene identifiers for a list of up to 10,000 genes. Additionally, the gene enrichment set tool uses the gene ID conversion to obtain the most similar genes in Arabidopsis to run Gene Ontology and metabolic pathway enrichment analysis in g:Profiler [22].

#### 2.2.6. Gene Expression Atlas

The MangoBase expression atlas contains 12 datasets and 80 experiments, representing some of the most significant mango RNA-seq experiments published to this date. Most of the data are from experiments that study mango fruit ripening and are based on unripe and ripe peel and pulp in several cultivars, showing different pulp firmness, sweetness, or peel coloration (Table 1). Additionally, several experiments have studied hot water postharvest treatment, and infection with *C. gloeosporioides*, and one of them provides seven experiments representing different mango tree organ tissues, which were used to annotate the ‘Alphonso’ genome. The MangoBase expression atlas has a menu with information about the datasets, where it is possible to obtain information about the experiments and their samples, including links to publications and raw data. As many of the available expression datasets were originally analyzed based on de novo transcriptome references, all the expression datasets included in MangoBase were reanalyzed using the ‘Tommy Atkins’ genome sequence as a reference, and all the data were normalized to transcripts per million (TPM).

Two tools are available in MangoBase for gene expression query: the Expression viewer and the Expression comparator. As an example of the use of the expression atlas, the word “SWEET” was used in the search box to find the SWEET sugar transporters as one of the responsible genes for increasing sugar content in mango fruits during ripening [11]. After finding many genes with the word sweet in their description, the InterPro domain “IPR004316: SWEET sugar transporter” was identified (Figure S1). Then, the word “IPR004316” was searched within the search box in the search result table, to find all genes with SWEET domains (Figure S2). Finally, 25 putative SWEET transporters were found. After evaluating their expression, six of them were selected to illustrate this example: Manin02g010170.1, Manin04g000720.1, Manin09g014730.1, Manin11g006170.1, Manin15g007950.1, and Manin16g006960.1. These genes were used as the input into the Expression viewer, choosing the “Tainong and Renong Pulp Ripening” dataset [11], which contains four stages of pulp ripening of two varieties, ‘Tainong’, with a high sugar content, and ‘Renong-1’, with a low sugar content.

**Figure 2:**
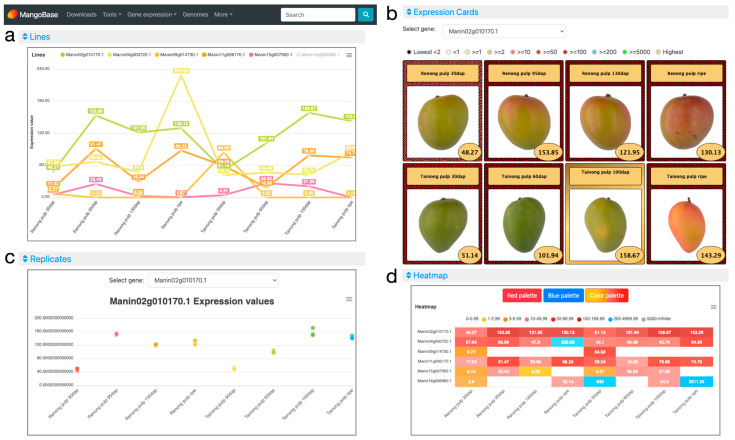
MangoBase gene expression atlas. (**a**) The Lines plot is useful to visualize and compare the expression of selected genes. (**b**) Expression cards showing experiment phenotypes of the selected gene. (**c**) The Replicates plot displays replicate expression values for each experiment of a selected gene. (**d**) Heatmap showing the expression of all genes and experiments grouped by expression range.

In the Lines plot (Figure 2a), it is possible to compare the expression of the six genes simultaneously. There, the gene Manin16g006960.1 showed a very high expression in the ripe stage in ‘Tainong’ (2911.55 TPM), and was deselected by clicking on its name on the legend of the plot to expand the lines of the rest of the genes. Then, it is easier to study the expression of the selected genes, which show peaks of expression in different fruit ripening stages. In the Expression Cards (Figure 2b), it is possible to select one gene to visualize its expression together with pictures showing the phenotype of the plant or parts of the plants used in the experiment. The colors of the cards represent different expression value ranges defined in the legend. The sample with the highest expression is highlighted in a golden card, and, in the case of samples below 2 TPM, the lowest expression values are highlighted in black cards. In the Replicates plot (Figure 2c), the expression of each replicate for a selected gene is displayed. In that way, we can explore if the replicates of the experiments have, as desired, a similar expression value or if, on the contrary, some replicates show a high variation. In the example (Figure 2c), we can observe that all the replicates have similar expression values, and, in many cases, their dots overlap on the plot. The Heatmap (Figure 2d) shows all genes and their experimental conditions simultaneously, to facilitate their comparison. Different ranges of expression are defined with different colors in the legend. Moving the cursor over each one of the color ranges in the legend will highlight the expression values of the samples within that range. In the example, among many other things, we can observe a high expression of Manin04g000720.1 in the ripe stage of ‘Reinong’, and an even higher expression for Manin16g006960.1 in ‘Tainong’ 30 days after pollination and in the ripe stages. Manin02g010170.1 shows a high expression in both mango accessions, ‘Renong’ and ‘Tainong’, especially 95 and 60 days after pollination, respectively. Finally, we can observe and download the expression values of the query genes in the Average values table, where genes show multiple annotations and are linked to their gene pages (Figure 1).

The gene Expression comparator also provides the results with similar visualization methods as the Expression viewer. The difference is that in the Expression comparator, any sample from any dataset can be combined for comparison, and one gene can be used for relative normalization to calculate fold-change or log-ratio values.

### 2.3. Gene Expression Data Clustering and Enrichment

**Figure 3:**
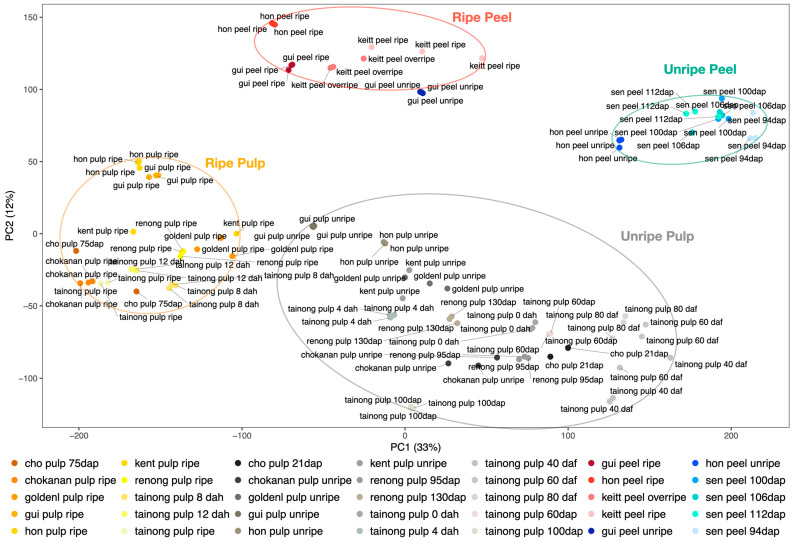
Principal component analysis (PCA) of mango expression data. Ripe pulp (grouped in an orange ellipse), ripe peel (in a red ellipse), unripe peel (in a green ellipse), and unripe pulp (grouped in a gray ellipse) samples are clustered in separated groups.

Mango pulp and peel experiments in control conditions, which represent most of the samples included in the expression atlas, were shown to be organized into four groups in a principal component analysis (PCA): ripe pulp, ripe peel, unripe pulp, and unripe peel (Figure 3). The unripe pulp and unripe peel contain multiple intermediate stages of ripening. Replicates from most of the experiments are grouped together. However, the unripe peel and pulp samples of the accession ‘Guire-82’ (“gui_peel_unripe” and “gui_pulp_unripe”) seem to be close to the ripe peel and ripe pulp experiments, respectively.

Considering the 80 experiments included in MangoBase expression atlas, 21,649 (81.33%) genes were expressed with two or more transcripts per million (TPM) of the total 26,618 genes predicted in the ‘Tommy Atkins’ mango genome. A total of 4969 (18.67%) genes were not expressed or expressed below 2 TPM (1548 with no expression, 5.82%, and 3421 with an expression between 0 and 2 TPM, 12.85%).

**Figure 4:**
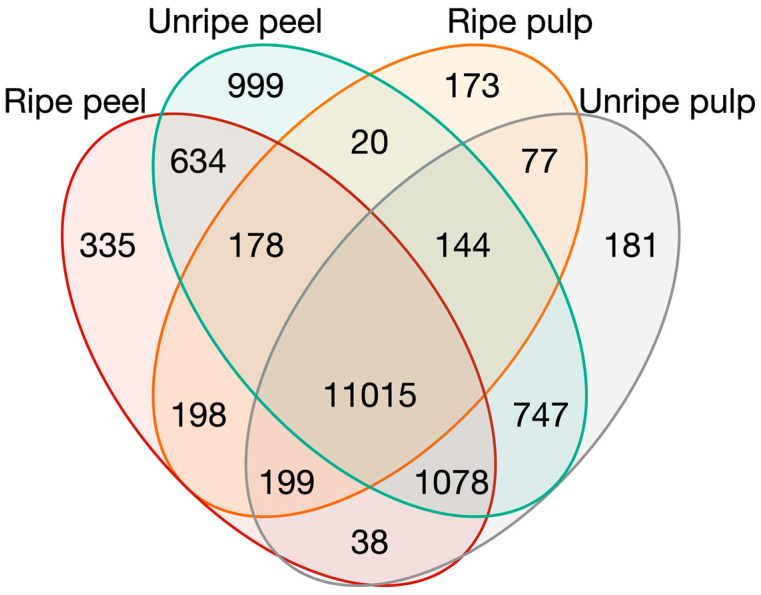
Venn diagram of genes expressed in ripe and unripe pulp and peel.

The experiments from the four groups defined in the PCA were compared in a Venn diagram (Figure 4) to find specific genes in ripe pulp, unripe pulp, ripe peel, and unripe peel. For this task, all samples in intermediate ripening stages were discarded, so only data from clearly ripe fruits or immature fruits were considered to identify specific genes of those stages. In the Venn diagram, 173, 181, 335, and 999 genes were classified as specific genes in ripe pulp, unripe pulp, ripe peel, and unripe peel, respectively. A total of 11,015 genes were found in all tissues, and 1078 genes were found in all tissues but ripe pulp, which also did not overlap with the rest of the groups in the first component of the PCA (Figure 1). On the other hand, there are 747 specific genes in common in unripe tissues, and 634 genes in the case of peel samples, much more than the 198 and 77 specific genes of ripe tissues and pulp, respectively. Specific genes for ripe pulp, unripe pulp, ripe peel, and unripe peel in control conditions and excluding intermediate ripening stages are available in Table S1.

The functions corresponding to the genes specifically expressed in the four groups were characterized through functional enrichment analysis of the biological processes associated with those genes (Figures S3–S6). In ripe pulp, terms related to sugar accumulation such as “glycolytic process”, or terms related to purine, and other terms related to plant hormones such as “cytokinin metabolic process,” are observed (Figure S3). In unripe pulp, there are terms related to development such as “cell wall organization and biogenesis” and “mitotic cell cycle” (Figure S4). Ripe peel specific genes show biological processes related to defense such as “response to fungus” or “response to jasmonic acid” (Figure S5). Finally, unripe peel includes terms related to responses to fungi and cuticle development such as “response to fungus” and “cutin biosynthetic process”, respectively (Figure S6). Enrichment results are available in Table S2.

## 3. Discussion

Mango genomic resources and expression data have increased significantly in recent years, with several genome sequences available and multiple experiments performed to study mango fruits. However, no platform dedicated to mango genomics, one of the most cultivated fruits in the world, is available to analyze and facilitate access to omics data. In order to fill this gap, we have developed MangoBase, a genomic portal for the genomic data of mango species and varieties. It includes multiple bioinformatics tools to explore gene expression data, compare sequences by similarity, and download sequences and annotations.

Most of the mango expression experiments have focused on fruit traits. The expression data available in the MangoBase expression atlas that included replicated fruit samples in control conditions were clustered in a principal component analysis (PCA). In the PCA, ripe pulp, unripe pulp, ripe peel, and unripe peel samples were clustered in four clearly separated groups. The unripe peel and pulp samples of the accession ‘Guire-82’ (gui_peel_unripe and gui_pulp_unripe) were positioned close to the ripe peel and pulp experiments, respectively. This accession has green mature mangoes, which might make the ripening stage identification difficult and might explain its position in the PCA, where it seems to be in a ripening stage closer to the other ripe fruits, and in between ripe and unripe samples.

Regarding the specific genes identified in the Venn diagram, a higher number of genes in unripe tissues and peel than in ripe tissues and pulp are observed. In the unripe stages, a fast fruit development takes place, due to cell division and enlargement, which might explain a higher activity than in already ripe fruits. On the other hand, the peel or exocarp, since it is in direct contact with the environment and is the visible part of the fruit for potential seed dispersal animals, might be involved in more processes than the mesocarp. Some of these processes might include changes in coloration, volatiles, attractors, defense against pathogens, defense against herbivory in unripe stages, avoiding desiccation, gas exchange, etc.

In the enrichment analysis of the exocarp (peel) and mesocarp (pulp) of ripe and unripe fruits, we can observe expected biological processes from the Gene Ontology (GO) involved in those tissues and conditions. For example, in the ripe pulp experiments, there are GO terms related to sugar accumulation such as “glycolytic process” and terms related to purine metabolism and plant hormones such as “cytokinin metabolic process”. Cytokinin expression has been also described in ripening kiwi fruits [25] and grapes [26]. In unripe pulp experiments, terms related to development such as “cell wall organization and biogenesis” and “mitotic cell cycle” are found, which are also expected since these experiments are comprehended by stages of growing fruits.

The terms related to pathogen defense found in peel, such as “response to fungus” might indicate that some of the fruits were exposed to fungi or the peel express these defense genes constitutively, or after priming, to be actively prepared to respond to fungi infection. On the other hand, jasmonic acid, referenced in the term “response to jasmonic acid,” has been described to be involved in resistance to fungi, but also in fruit growth and other processes such as fruit coloration and softening [27,28].

Additionally, the unripe peel specific genes are enriched in terms related to cuticle development such as “cutin biosynthetic process”. The cuticle is a hydrophobic layer, composed mostly of cutin and waxes, which is an important constituent of the exocarp. This layer, synthesized by epidermal cells, is the most external barrier between the fruit and the environment and has important functions, such as limiting water loss and gas diffusion, and providing protection against insects, pathogens, and ultraviolet radiation [12].

Some terms related to root development and morphogenesis in unripe peel could be explained by common functions in development that are assigned to Gene Ontology terms of genes expressed in roots, but also expressed in other tissues.

MangoBase provides a starting point for accessing mango genomic data and a reference point for integrating new data and tools in the future. Our efforts aim to involve the mango scientific community into MangoBase in order to integrate the multiple currently available and developing mango genomes and unify the gene annotations consensually. In the future, our plans include the integration of a large amount of genetic variation data with tools that allow easy visualization and identification and their possible effects on the coding sequence and nearby regulatory regions. In addition, we hope that all these data will allow the generation of a pangenomic reference for mango that integrates the synteny and genetic variation of multiple *Mangifera* species and accessions.

## 4. Materials and Methods

### 4.1. Genomics Portal Implementation

MangoBase was implemented using EasyGDB [16]. The code used to customize the genomic portal is available on GitHub (https://github.com/noefp/mangobase (accessed on 8 March 2023)). ‘Tommy Atkins’ genes were annotated with InterProScan, and diamond BLASTp [29] best hit with the databases Araport 11, SwissProt, and trEmbl.

### 4.2. Gene Expression Atlas Data Analysis

Gene expression datasets included in the MangoBase expression atlas were selected from already published mango RNA-seq experiments (Table 1). Raw reads were downloaded from the NCBI Sequence Read Archive BioProjects PRJNA487154, PRJNA487154, PRJNA258477, PRJNA253272, PRJNA304093, PRJNA629065, PRJNA697524, PRJNA803945, PRJNA515564, PRJNA227243, PRJNA286253, and PRJNA575336. SRA Explorer (https://sra-explorer.info/ (accessed on 8 March 2023)) was used to download the raw reads in a compressed fastq file format. Then, Trimmomatic v 0.39 [30] was used to remove adapter sequences and low-quality reads, with the options ILLUMINACLIP:TruSeq3-PE.fa:2:30:10 LEADING:3 TRAILING:3 SLIDINGWINDOW:4:15 MINLEN:36. Raw and processed reads were inspected with FastQC (https://www.bioinformatics.babraham.ac.uk/projects/fastqc (accessed on 8 March 2023)) and MultiQC [31]. Processed reads were mapped to the ‘Tommy Atkins’ mango genome sequence vTA4 [3] using Hisat2 v2.2.1 [32], and converted to sorted BAM files with Samtools v.1.13 [33]. Gene counts were calculated with FeatureCounts from the Subread package v.2.0.3 [34], and then normalized to transcripts per million (TPM) using R function convertCounts from the package DGEobj.utils.

### 4.3. Gene Expression Data Clustering and Enrichment Analyses

Principal component analysis (PCA), specific gene identification, and the enrichment analyses were conducted in R v.4.2.1. The experiment replicates were clustered in a PCA plot using logarithmic values in the prcomp function included in stats v.4.2.1. Genes with a minimum value of 2 transcripts per million (TPM) from experiments of fruit pulp and peel in control conditions were used in the specific gene analysis. Samples with intermediate ripening stages were discarded to avoid overlapping between the ripe and immature stages. The functional enrichment was done with clusterProfiler v.4.4.4 package using the most similar protein found in the *Arabidopsis thaliana* Araport11 protein set and Diamond BLASTp v.2.0.14 [29] filtered with a minimum score of 45. For the PCA, gene counts were filtered using a minimum of 1 CPM in 1 library, and then normalized to trimmed mean of M-values (TMM) using edgeR v.3.38.4 [35].

### 4.4. Gene Lookup and Gene Enrichment Set Tools

The most similar genes between each of the available mango protein sets and between ‘Tommy Atkins’ and Arabidopsis Araport11 were calculated using Diamond BLASTp v.2.0.14 [29] with the options—very-sensitive and —max-target-seqs 1. Later, the most similar hits found in both directions were merged and hits with a score value lower than 45 were filtered out.

## Figures and Tables

**Table 1:** Expression datasets available at MangoBase.

Dataset Name	Experiment Number	Description	Publication
‘Alphonso’ multiple tissues	7	Root, bark, leaf, flower, peel, pulp, and seed in control conditions used for the annotation of the ‘Alphonso’ genome	[5]
Pulp and peel ripening	16	Pulp and peel ripening of ‘Hongyu’, ‘Guire-82’, and ‘Sensation’, which show different coloration over maturation	[5]
‘Chokanan’ pulp ripening	2	Pulp ripening in ‘Chokanan’	[9]
‘Chokanan’ and ‘Golden phoenix’ pulp ripening	4	Pulp ripening of varieties showing different pulp firmness	[10]
‘Kent’ pulp ripening	2	Pulp ripening in ‘Kent’	[8]
‘Tainong’ and ‘Renong’ pulp ripening	8	Time series of pulp ripening of two varieties with different fruit sweetness	[11]
‘Tainong’ pulp ripening	8	Time series of pulp ripening	[23]
‘Keitt’ peel ripening	2	Peel ripening in ‘Keitt’	[12]
‘Keitt’ peel storage	7	Peel response to storage in low temperatures	[13]
‘Shelly’ peel hot water treatment	8	Time series of peel response to hot water treatment	[14]
‘Ataulfo’ pulp quarantine postharvest treatment	4	Pulp ripening quarantine postharvest treatment	[24]
‘Shelly’ peel *Colletotrichum gloeosporioides* treatment	12	Time series of peel in response to *C. gloeosporioides*	[15]

## Data Availability

Mango genomic data was retrieved from the NCBI (https://www.ncbi.nlm.nih.gov/data-hub/genome/GCF_011075055.1/ (accessed on 8 March 2023)) and the CNCB (GWHABLA00000000). Tommy Atkins data were obtained from the Mango International Consortium [3]. Gene expression data were retrieved from the Sequence Read Archive from the BioProjects: PRJNA487154, PRJNA487154, PRJNA258477, PRJNA253272, PRJNA304093, PRJNA629065, PRJNA697524, PRJNA803945, PRJNA515564, PRJNA227243, PRJNA286253, and PRJNA575336. The genomic portal is accessible at https://mangobase.org/ (accessed on 1 January 2023), and the code used to customize the site is available in Github (https://github.com/noefp/mangobase, accessed on 1 January 2023).

## Supplementary Materials

The following supporting information can be downloaded at: https://www.mdpi.com/2223-7747/12/6/1273/s1, Figure S1: Result of searching “SWEET” in MangoBase; Figure S2: Filtering by the term “ IPR004316” in the search results to identify all genes in MangoBase containing the protein domain “SWEET sugar transporter”; Figure S3: Functional enrichment of biological processes of ripe pulp specific genes; Figure S4: Functional enrichment of biological processes of unripe pulp specific genes; Figure S5: Functional enrichment of biological processes of ripe peel specific genes; Figure S6: Functional enrichment of biological processes of unripe peel specific genes; Table S1: specific genes; Table S2: enrichment.
