# Supplementary figures and images for: MangoBase: A Genomics Portal and Gene Expression Atlas for Mangifera indica
Source: Plants (Basel). 2023 Mar 10;12(6):1273. doi: 10.3390/plants12061273 (PMC10058708; doi:10.3390/plants12061273)

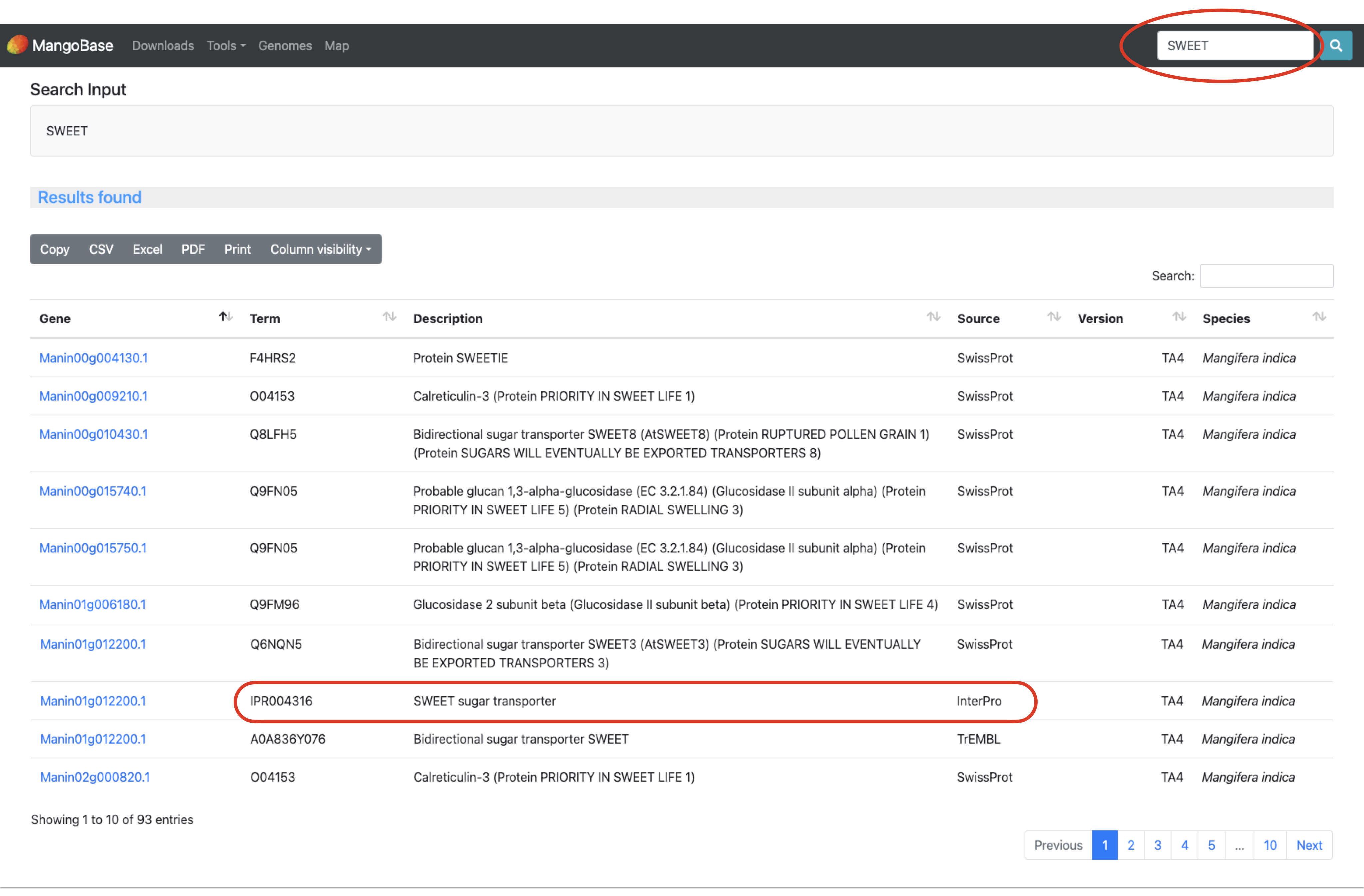

Supplement: Supplementary file 1 [file plants-12-01273-s001.zip › Figure S1 - MangoBase use case search.png]

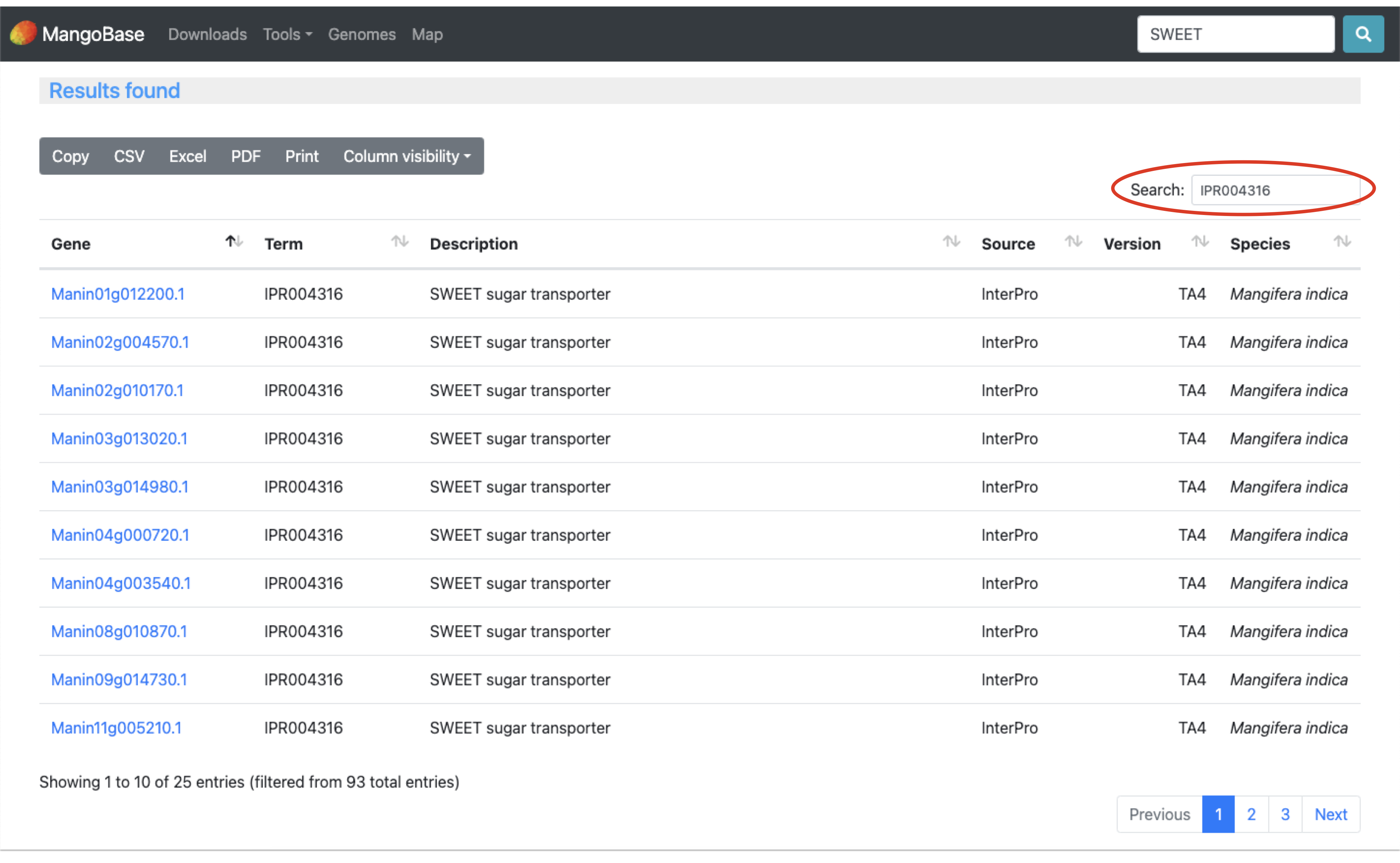

Supplement: Supplementary file 1 [file plants-12-01273-s001.zip › Figure S2 - MangoBase use case search filtering.png]

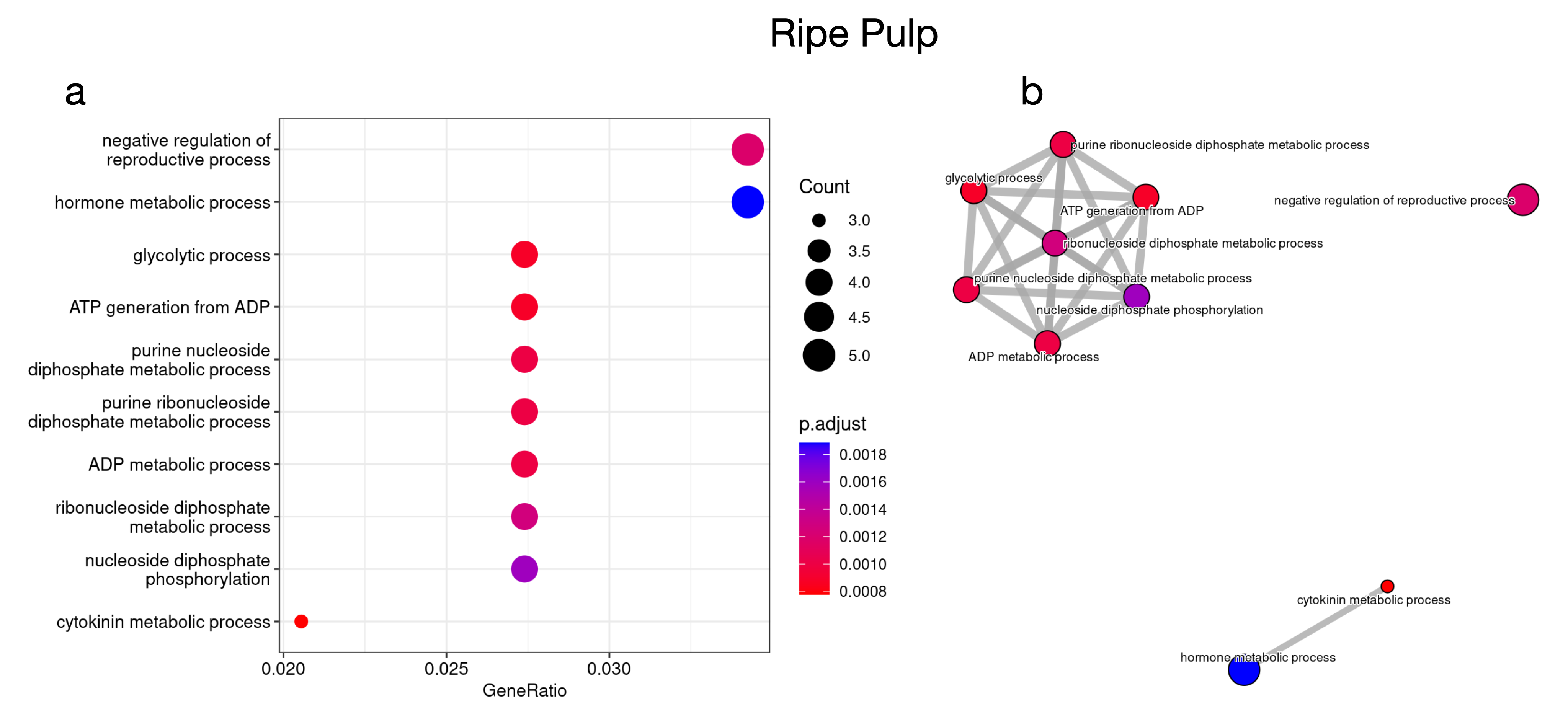

Supplement: Supplementary file 1 [file plants-12-01273-s001.zip › Figure S3 - Ripe pulp BP enrichment.png]

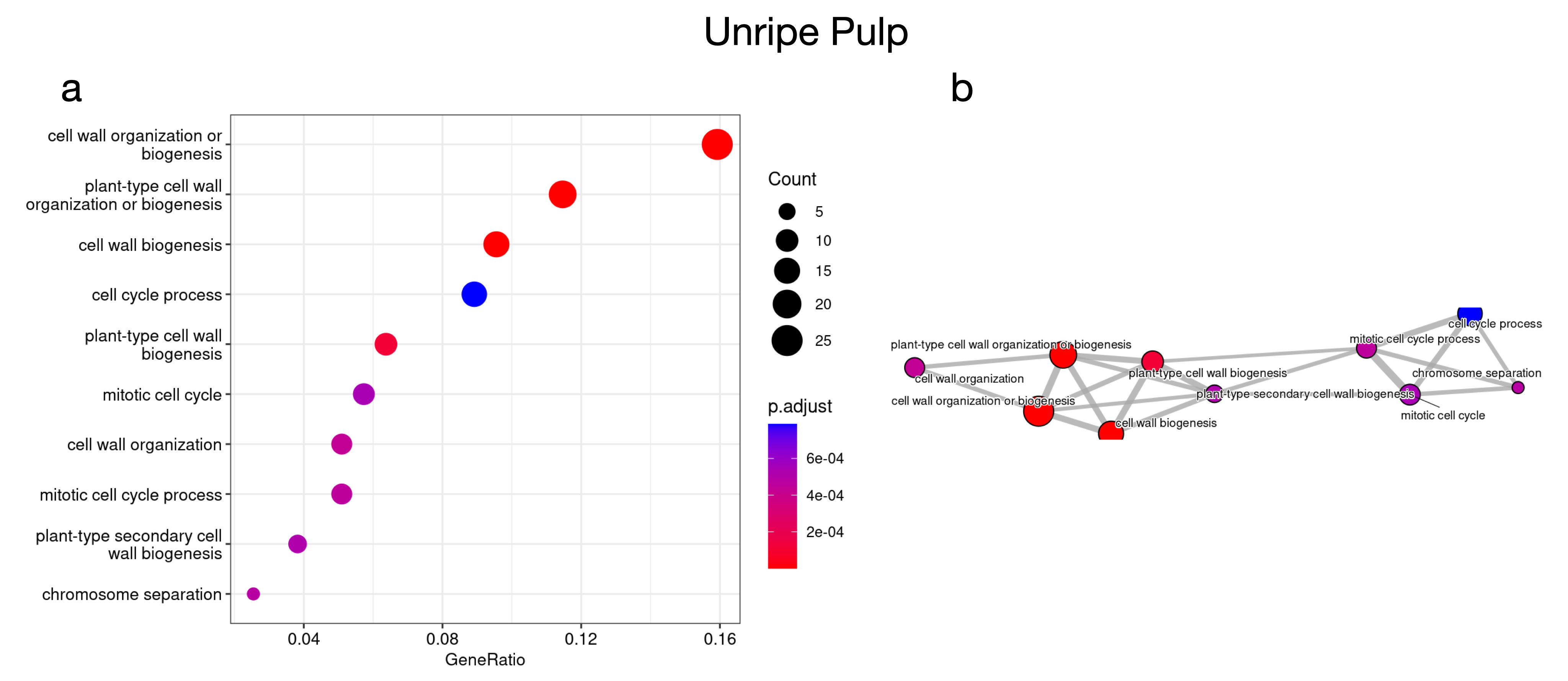

Supplement: Supplementary file 1 [file plants-12-01273-s001.zip › Figure S4 - Unripe pulp BP enrichment.png]

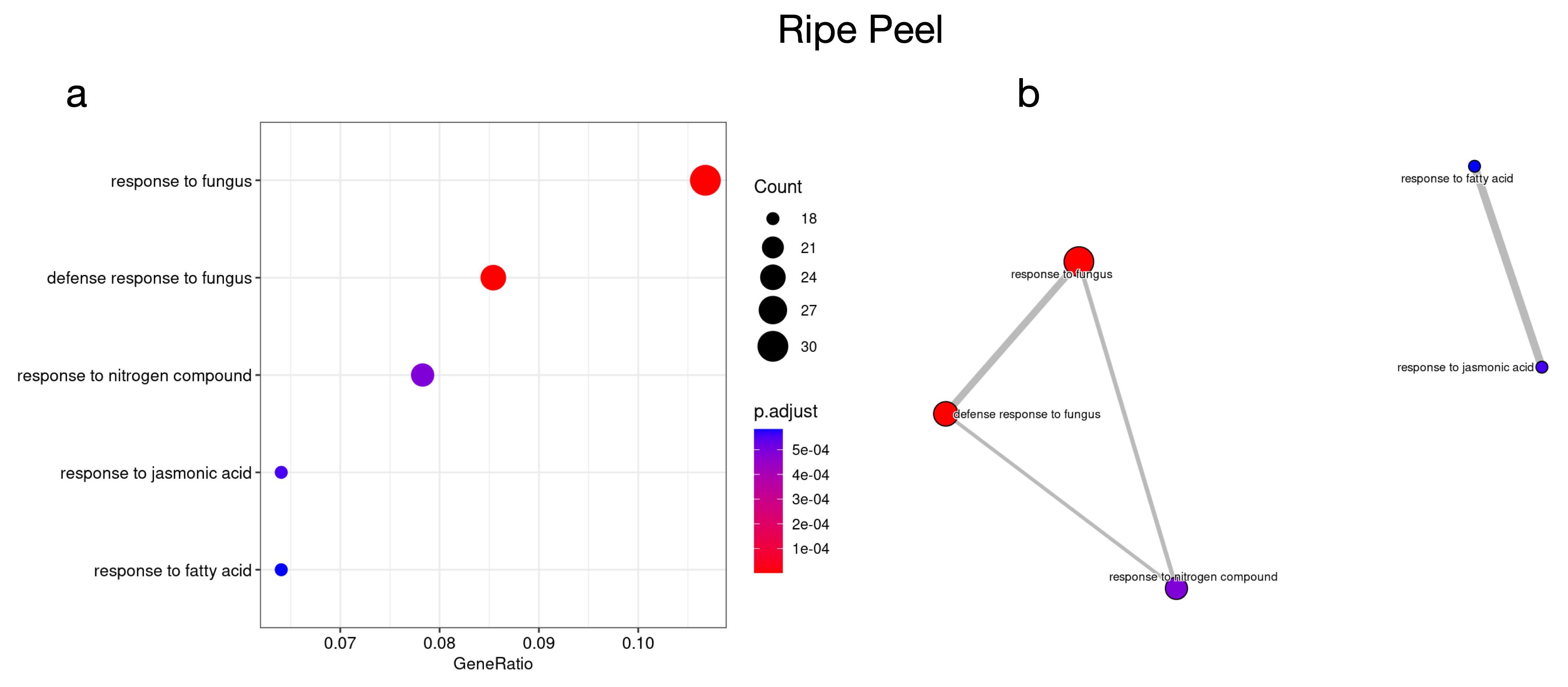

Supplement: Supplementary file 1 [file plants-12-01273-s001.zip › Figure S5 - Ripe peel BP enrichment.png]

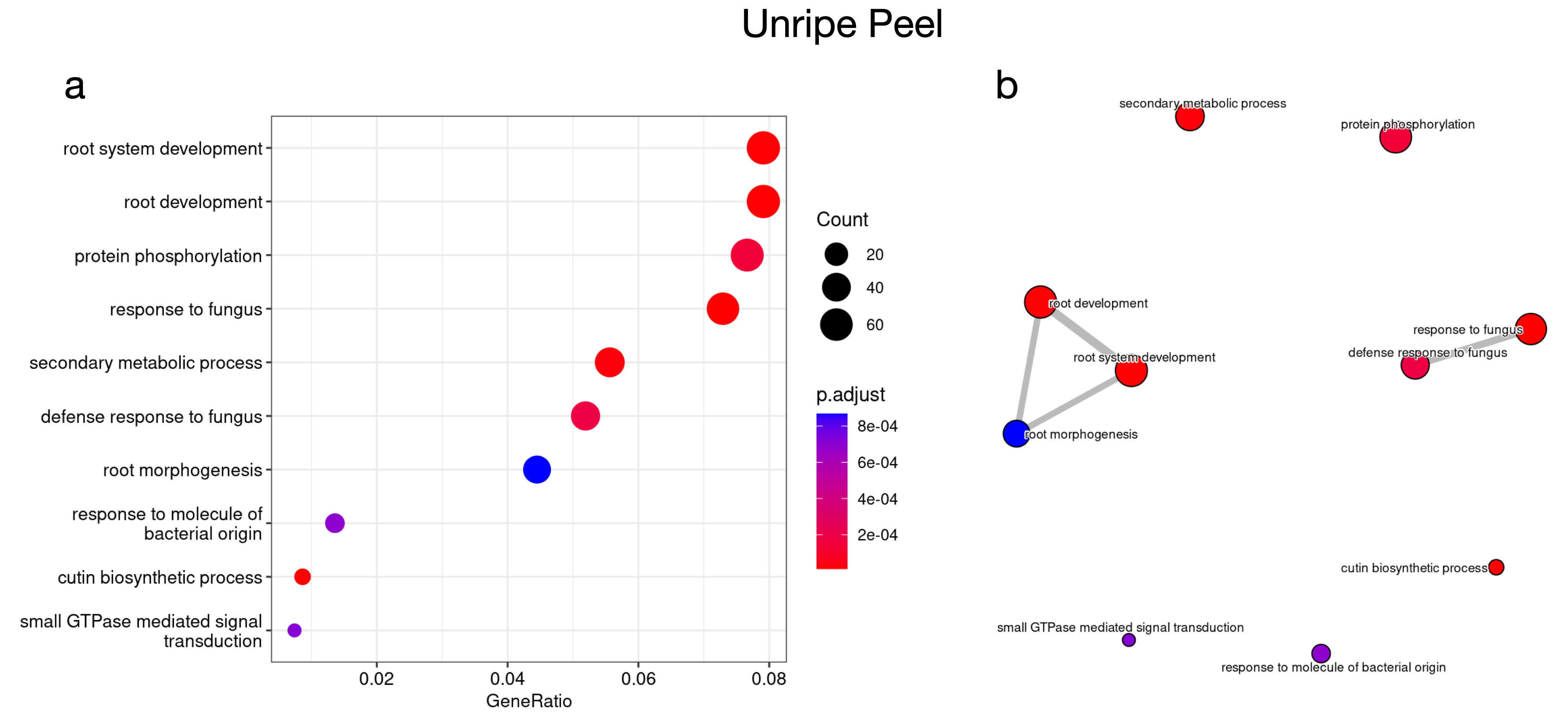

Supplement: Supplementary file 1 [file plants-12-01273-s001.zip › Figure S6 - Unripe peel BP enrichment.png]
